# Ginsenosides: A Potential Neuroprotective Agent

**DOI:** 10.1155/2018/8174345

**Published:** 2018-05-08

**Authors:** Mengmeng Zheng, Yizhou Xin, Yujuan Li, Fangxue Xu, Xiaozhi Xi, Hong Guo, Xiaowei Cui, Hui Cao, Xi Zhang, Chunchao Han

**Affiliations:** ^1^School of Pharmacy, Shandong University of Traditional Chinese Medicine, Jinan 250355, China; ^2^The Affiliated Hospital of Shandong University of Traditional Chinese Medicine, Jinan 250011, China

## Abstract

Ginseng is a traditional Chinese medicine with a wide range of pharmacological activities. Ginsenosides are the major constituents of ginseng. Ginsenosides have the unique biological activity and medicinal value, such as antitumor, anti-inflammatory, antioxidation, and inhibition of cell apoptosis. With the increase of stress in life, the incidence of nervous system diseases is also increasing. Neurological diseases pose a huge burden on people's life and health. In recent years, some studies have shown that ginsenosides have a certain role in the prevention and treatment of neurological diseases. However, the research is still in its infancy, and the relevant mechanisms are complex. In the paper, we review the effects and mechanisms of ginsenosides on epilepsy, depression, cerebral ischemia reperfusion injury, Alzheimer's disease, and Parkinson's disease. We hope to provide a theoretical basis for the treatment of nervous system diseases by ginsenosides.

## 1. Introduction

**Figure 1:**
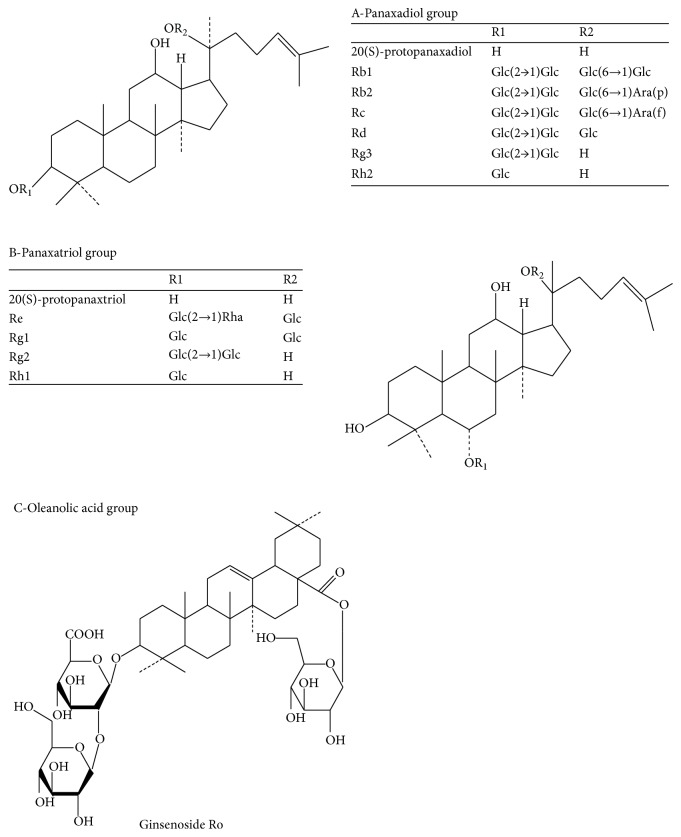
Chemical structure of Panaxadiol, Panaxatriol, and Oleanolic acid group.

Ginseng is the traditional valuable medicinal herb that has been widely used in China for thousands of years. Ginseng is used as energy booster, and it can fortify the spleen to benefit the lungs, nourish fluids, calm the heart, tranquilize the mind, and so on [1–3]. Ginseng has such a good effect, mainly due to ginsenosides. Ginsenosides are the major active ingredients of ginseng and are extracted from roots, fruits, stems, and leaves of ginseng. At present, more than 60 ginsenosides were isolated and identified from Araliaceae family. The basic structure of ginsenosides is similar because almost all ginsenosides contain 30 carbon atoms, and they are arranged in four rings of steroid nuclei [4]. According to the difference in the position and quantity of sugar moiety, ginsenosides are divided into three types (Figure 1): A-Panaxadiol group (e.g., Rb1, Rb2, Rb3, Rc, Rd, Rg3, and Rh2), B-Panaxatriol group (e.g., Re, Rg1, Rg2, and Rh1), and C-Oleanolic acid group (e.g., Ro) [5, 6]. Each ginsenoside plays a different pharmacological role. Modern pharmacological experiments have shown that ginsenosides have many biological activities. For example, ginsenosides have good effects in anticancer, anti-inflammation, antioxidation, antiaging, antifatigue, and physiological functions [7–10].

The main features of nervous system disease are sensation, movement, consciousness, and autonomic nervous dysfunction. Common neurological disorders include Alzheimer's disease, Parkinson's disease, epilepsy, and depression. Nervous system diseases have a great impact on the life and work of the patients and can even lead to life-threatening situations. Nervous system diseases have become another major disease after heart disease and cancer. With the increase of aging population and the pressure of life, the prevalence of nervous system diseases will become higher and higher. Therefore, it is very meaningful to study and treat nervous system diseases. Recently, ginsenosides are becoming more and more concerned in the treatment of neurological diseases. Some studies have shown that ginsenosides do have good preventive and therapeutic effect on neurological diseases [11, 12]. For instance, ginsenoside Rg2 protects against memory impairment via antiapoptosis in rat models with vascular dementia [13]. The neuroprotective mechanisms of ginsenosides include antioxidant effect, antiapoptosis effect, estrogen-like effect, restraining the influence of nitric oxide and nitric oxide synthase, and improving mitochondrial dysfunction [14].

**Figure 2:**
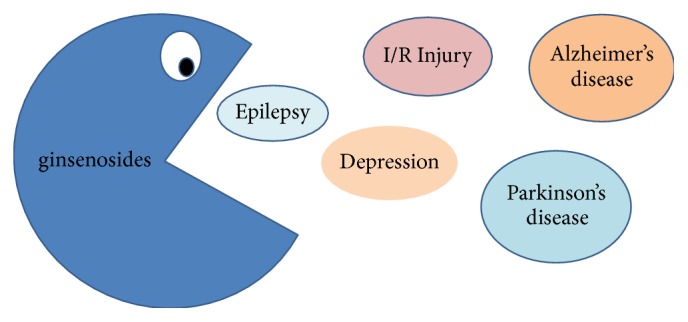
The effect of ginsensides on nervous system disease, mainly including epilepsy, depression, cerebral ischemia reperfusion injury, Alzheimer's disease, and Parkinson's disease.

**Figure 3:**
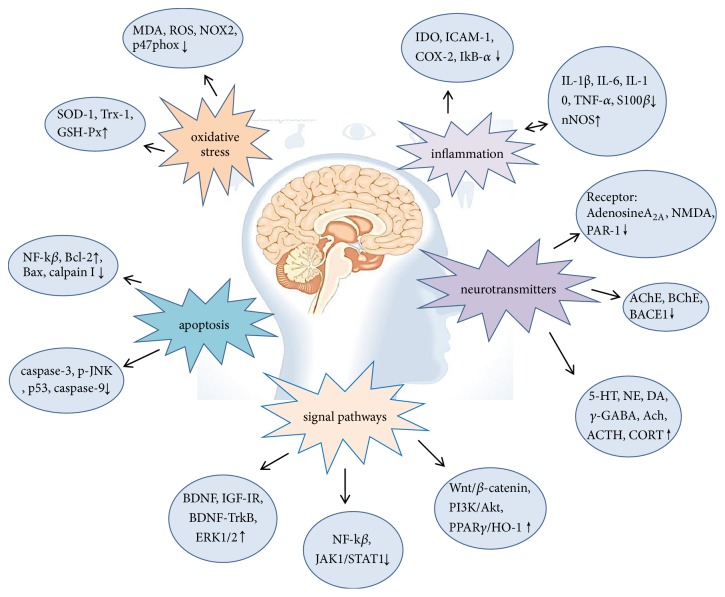
Several neuroprotection mechanisms of ginsenosides.

At present, the treatment of nervous system diseases is mainly drugs and surgical treatment. However, patients often cannot adhere to the formal medication. In addition, long-term use of drugs can lead to drug accumulation and poisoning. Surgical treatment often increases the chance of infection and leads to other dysfunctions. In recent years, western countries have been using natural medicine to treat nervous system diseases, which is considered to be promising research method [15]. Ginsenoside is one of the most valuable natural medicines. Animal experiments have shown that ginsenosides are effective in treating nervous system diseases, such as Parkinson's and Alzheimer's disease (Figure 2). In the paper, we reviewed the mechanism of ginsenosides in Parkinson's disease, Alzheimer's disease, epilepsy, cerebral ischemia reperfusion injury, and depression in order to provide assistance for the treatment of nervous system diseases by ginsenosides and stimulate more research to examine the clinical efficacy of ginsenosides (Figure 3).

## 2. The Pharmacological Activity and Mechanism of Ginsenosides in Nervous System Diseases

### 2.1. Antiepilepsy Effect

Epilepsy is a kind of chronic brain disease caused by abnormal discharge of neurons in the brain, which is characterized by recurrent seizures [16]. There are about 65 million people with epilepsy in the world, and 2.4 million new cases occur every year [17]. Clinically, the treatment of epilepsy is mainly antiepileptic drugs, but antiepileptic drugs still cannot effectively control many epileptic patients. Antiepileptic drugs also could lead to death, neurological disorders, and other adverse reactions. Therefore, it is meaningful to find new antiepileptic drugs. Happily, natural products usually have less toxic and side effects. Ginsenosides are the main active components of ginseng and have a good antiepileptic effect.

Lian et al. have compared the anticonvulsant activity of whole root extract, whole leaves/stems extract, and Rb extract. Three seizure models were prepared by kainic acid (KA), pilocarpine, and pentylenetetrazol (PTZ), respectively. The results showed that Rb extract had obvious anticonvulsant effect and was dose-dependent in three models. The Rb extract not only could increase the latency to the seizures, but also could decrease the seizure score, weight loss, the seizure duration, and neuronal damage. The above results suggest that panaxadiol group has a good effect on nervous system, because the Rb component belongs to panaxadiol group [18]. Compared with the Rb extract, the root extract and the leaves/stems extract had no significant anticonvulsant effect. This hints that some ginsenosides have anticonvulsant effects, while some ginsenosides do not have anticonvulsant effects. Lian et al. also compared the anticonvulsant effects of ginsenosides Rb1, Rb3, Rd, and mixtures of them. The results suggest that the mixtures of purified ginsenosides Rb1, Rb3 with or without Rd has significant anticonvulsant effects in three models induced by KA, PTZ, and pilocarpine. Amusing, no one individual ginsenoside accounted for the activity of the Rb extract. Therefore, it is of great significance to explore the combination of ginsenosides for the development of anticonvulsant drugs. Furthermore, studies have shown that ginsenoside Rb2 plays an important role in anticonvulsant activity [19]. These results suggest that the Rb fraction has the potential to become anticonvulsant drug.

Intracellular calcium concentration Ca^2+^ is an important indicator of neurological disorder. The level of Ca^2+^ would increase in epilepsy. Total ginsenosides and ginsenoside Rg3 could inhibit the increase of Ca^2+^ induced by Mg^2+^. Ginsenosides could modulate perturbed homeostasis of Ca^2+^ by the inhibition of N-methyl-D-aspartate receptor [20]. Besides, oxidative stress could lead to hippocampal degeneration. It is interesting to note that ginsenosides attenuated oxidative stress in the synaptosome and reduced synaptic vesicles at the presynaptic terminals dose dependently. What is more, not adenosine A_1_ receptor or adenosine A_2B_ receptor, but adenosine A_2A_ receptors play an important role in antiepilepsy. Therefore, ginsenosides exert antiepilepsy effect by activating adenosine A_2A_ receptors [21] (Table 1).

### 2.2. Antidepressant-Like Effect

Depression is neuropsychiatric disorder characterized by sleep disturbances, low self-esteem, guilty feelings, and suicidal tendencies [22–24]. Depression can reduce the body weight and appetite of patients [25]. About 15% people with depression died from suicide. According to the World Health Organization, depression has become the second major disease burden in China. Clinically, monoamine oxidase inhibitors are commonly used to treat depression [26]. The mechanism of antidepressant drugs may be related to proteins, neurotransmitters, hormones, energy sources, and minerals [27]. Nevertheless, antidepressant drugs would cause irreversible side effects. Therefore, it is necessary to find a safe and effective medicine for treating depression. It is wise to look for active ingredients from natural products.

In order to explore the antidepressant effect of ginsenoside Rb3, some tests and biochemical changes were carried out. Researchers found that ginsenoside Rb3 could significantly reduce immobility time in forced swim test and tail suspension test. In addition, ginsenoside Rb3 decreased the number of escape failures in the learned helplessness procedure. Besides, ginsenoside Rb3 not only increased sucrose preference, locomotor activity, and novelty-suppressed of mice, but also attenuated hypothermia, palpebral ptosis, and akinesia. Furthermore, ginsenoside Rb3 would reverse the decrease of hippocampal weight and the brain-derived neurotrophic factor (BDNF) level in the hippocampus [28]. As we all known, substances can be hydrolyzed in the intestine. In order to explore substances that exert antidepressant activity, ginsenoside Rb3 and its four-deglycosylated derivatives (ginsenoside Rg3, ginsenoside Rh2, compound K, and 20(S)-protopanaxadiol (PPD)) were tested in depression mice model. The results indicated that ginsenoside Rg3 had better antidepressant effect than compound K, ginsenoside Rb3, ginsenoside Rh2, and PPD. Ginsenoside Rg3 exerted antidepressant effect by regulating noradrenaline (NA), adrenocorticotropic hormone (ACTH), and corticosterone (CORT) levels in the brain region of mice. Moreover, the effects of compound K and ginsenoside Rb3 were similar, but ginsenoside Rh2 and PPD failed to show any effect [29]. In addition, ginsenoside Rg3 also exerted antidepressant effect through promoting the hippocampal BDNF signaling pathway [30].

The antidepressant effect of ginsenoside Rg1 was explored in a mice model of depression and a mouse model of chronic mild stress (CMS) depression. Changes were investigated in hippocampal neurogenesis, spine density, BDNF signaling pathway, and serum corticosterone level. The results suggested that ginsenoside Rg1 reversed the decrease of dendritic spine density and hippocampal neurogenesis. Moreover, ginsenoside Rg1 exerted antidepressant effect by activating the BDNF signaling pathway and upregulating hippocampal neurogenesis [31]. In addition, ginsenoside Rg1 could increase protein kinase A (PKA) and cAMP-response element binding protein (CREB) phosphorylation levels. And ginsenoside Rg1 increased BDNF expression in the amygdala of rats [32]. These results suggest that ginsenoside Rb1 is a very promising antidepressant. Moreover, depression-like and anxiety-like behaviors and cognition memory deficit were prepared by repeated immobilization. Ginsenoside Re could decrease the level of tyrosine hydroxylase (TH) expression in the locus coeruleus and increase BDNF mRNA expression in the hippocampus. What is more, ginsenoside Re significantly restored body weight and serum corticosterone (CORT) level and blocked the increase of corticotrophin-releasing factor (CRF) in the hypothalamus [33]. So, ginsenoside Re inhibited the stress-induced behavioral deficits by modulating the central noradrenergic system. It is well known that menopausal women are more likely to suffer from depression and the depression is more severe. The findings indicated that ginsenoside Rb1 and compound K significantly prevented ovariectomy-induced prolongation of immobility, whereas PPD, ginsenosides Rg1, and Ro did not have any significant effect on immobility time. In addition, coadministration of ginsenoside Rb1 and ritanserin, 5-HT_2A_ receptor antagonist, did not reduce the immobility time in ovariectomized mice, because ritanserin antagonized the effect of ginsenoside Rb1. Therefore, ginsenoside Rb1 activated 5-HT_2A_ receptor to exert antidepressant effect [34].

Antidepression effect of St John's Wort extract (SJ), ginsenosides, and clomipramine (CPM) were observed in chronic unpredictable mild stress-induced depression by using a combination of behavioral assessments and metabonomics. Ginsenosides could increase the activity frequency of model animals, while SJ and CPM had only a mild effect. Besides, ginsenosides not only significantly increased food consumption and body weight, but also alleviated the reduction of adrenal and thymus indices. Furthermore, ginsenosides significantly increased plasma ACTH and serum CORT levels in model rats [35]. Kang et al. came up with a new idea that the antidepression effect of total ginsenosides may be associated with its anti-inflammation. The results suggested that ginsenosides would attenuate LPS-induced depression-like behavior in forced swimming test, tail suspension test, and sucrose preference test. On the one hand, total ginsenosides decreased 5-HT level, tryptophane turnover, and indoleamine 2, 3-dioxygenase (IDO) activities in brain. On the other hand, ginsenosides reduced the levels of IL-1*β*, IL-6, TNF-*α*, and IDO in hippocampus. Therefore, studying the peripheral anti-inflammatory effects of ginsenosides may contribute to treatment of neurological diseases [36] (Table 1).

### 2.3. Protection against Cerebral Ischemia Reperfusion Injury

Acute cerebral ischemia can cause serious nervous system and motor impairment, threatening human life and health. It has become an important disease, which endangers the health of the elderly in modern society. Cerebral ischemia reperfusion injury can lead to apoptosis and necrosis in the ischemic and peripheral nerve cells. Cerebral ischemia reperfusion injury may be the underlying pathological cause of stroke, which is one of leading causes of death and acquired disability in the world [69, 70]. In addition, the incidence of stroke is higher in developing countries [71]. The high mortality and disability rates of ischemic stroke are a huge burden on society and family. There are few methods to treat ischemia reperfusion injury, and the mechanisms involved are very intricate. Ginsenosides play an important role in the treatment of cerebral ischemia reperfusion injury. Some studies have explored the mechanism of ginsenosides in the treatment of cerebral ischemia reperfusion injury.

At present, bone marrow mesenchymal stem cell (BMSC) transplantation has applied to the treatment of cerebral ischemia disease. Nevertheless, the method also has some drawbacks, such as low neural cell conversion rate and weak proliferation ability [37]. Ginsenoside Rg1 is considered to be able to treat cerebral ischemia because its molecules are small enough to pass through the blood-brain barrier [38]. Ischemia reperfusion would cause significant nervous deficit, increase the brain water content, and infarct volume. Interesting, ginsenoside Rg1 can improve nervous deficit, reduce cell apoptosis, and increase the neuron-specific enolase positive cells and glial fibrillary acidic protein positive cells. Furthermore, ginsenoside Rg1 not only significantly increased Bcl-2 protein level, but also decreased Bax protein level. Combined application of ginsenoside Rg1 and BMSC transplantation promoted brain tissue repair through enhancing neuron-like cell differentiation and the antiapoptosis effect of ginsenoside Rg1 in cerebral ischemia reperfusion injury [37]. Ginsenoside Rg1 also improved neurological injury via downregulating aquaporin 4 expression [38]. It is well known that inflammation and neuronal apoptosis occur frequently in cerebral ischemia reperfusion injury. Peroxisome proliferator-activated receptor *γ* (PPAR*γ*) could mediate many signaling pathways in various pathological conditions [72]. Heme oxygenase-1 (HO-1) is one of the downstream effecter of PPAR*γ* [73]. PPAR*γ*/HO-1 signaling can inhibit apoptosis and inflammation. The relationship of ginsenoside Rg1 and PPAR*γ*/HO-1 signaling was studied in cerebral ischemia reperfusion injury. They found that the effects of ginsenoside Rg1 and rosiglitazone, the agonist of PPAR*γ*, were similar in activating the PPAR*γ*/HO-1 signaling. Besides, ginsenoside Rg1 not only decreased the levels of IL-1*β*, TNF-*α*, and high-mobility group box-1 (HMGB1), but also downregulated expressions of cleaved caspase-3, cleaved caspase-9, and receptor for advanced glycation end product (RAGE) in model rats [39]. After treatment of ginsenoside Rg1, the brain infarct volume and the permeability of blood-brain barrier were deceased in ischemia brain. And ginsenoside Rg1 would downregulate the protein and mRNA levels of protease-activated receptor-1 (PAR-1). As PAR-1 expression rises, so does blood-brain barrier permeability [40]. What is more, astrocytes play an important role in the ischemic neuronal cell death. Although ginsenoside Rg1 did not change the viability of astrocytes, ginsenoside Rg1 could attenuate apoptosis and inhibit intracellular Ca^2+^ overload in astrocytes. Besides, ginsenoside Rg1 could reduce the loss of mitochondrial membrane potential and ROS production in astrocytes [41].

Local inflammation may aggravate cerebral ischemia reperfusion injury. Study showed that ginsenoside Rb1 reversed the activation of nuclear factor kappa B (NF-k*β*) signaling pathway and the elevation of TNF-*α* and IL-6 in the ischemic hemisphere [43]. As far as we know, intranasal administration could effectively improve the bioavailability of drug. The concentration of ginsenoside Rb1 could achieve peak concentration at early time after intranasal administration. Moreover, ginsenoside Rb1 could inhibit the increase of Beclin 1 level, which is the index of autophagy activity [44]. To explore whether ginsenosides Rg1 and Rb1 have synergistic effect on neuroprotection in ischemia reperfusion, ginsenosides Rg1, Rb1, and Rg1/Rb1 were studied. The results showed that ginsenosides Rg1 and Rb1 significantly improved neurobehavioral deficits via reducing the cell apoptosis and alleviating the lipid peroxidation in the middle cerebral artery occlusion (MCAO) group. Moreover, ginsenosides Rg1 and Rb1 could reverse the decrease of thioredoxin-1 and superoxide dismutase levels, and they could improve the expression of heat shock protein 70 (HSP70), Akt, and the p-NF-kB p65 subunit in the MCAO group. However, ginsenosides Rg1/Rb1 did not have a synergistic effect [45]. Therefore, it may be best to use ginsenosides Rg1 or Rb1 alone for treating ischemic stroke. In contrast, astragaloside IV combined with ginsenosides Rg1, Rb1, and notoginsenoside R1 have greater effect than those of effective components alone. The combination of four active substrates exerted protective effects on cerebral ischemia injury via antiapoptosis and anti-inflammation. The mechanisms may be associated with inhibiting the activation of NF-k*β*, tyrosine kinase 1/signal transducer, and activator of transcription-1 signal pathways and regulating endoplasmic reticulum stress after cerebral ischemia [42].

Ginsenoside Rd is thought to have neuroprotective effect via antioxidant [74]. Ginsenoside Rd protects against cerebral ischemia through several pathways. Some researches proved that ginsenoside Rd exerted the neuroprotective effect by promoting neurogenesis, increasing vascular endothelial growth factor (VEGF) and BDNF expression, and activating the PI3K/Akt and ERK1/2 pathways [46]. Besides, ginsenoside Rd can improve behavior score and viability of neurons by inhibiting the hyperactive phosphorylation of N-methyl-d -aspartate receptor 2B (NMDAR 2B) subunit and decreasing its expression levels in cell membrane of ischemia-reperfusion injury models [47]. The content of ginsenoside Re is the highest among ginsenosides and has good activity. Ginsenoside Re also has protective effect on cerebral ischemia reperfusion injury. Ginsenoside Re exerted the neuroprotection via significantly decreasing the malondialdehyde (MDA) content and increasing the activity of H^+^-ATPase [48]. Besides, some experiments suggested that ginsenoside Rg3 modulated various types of ion channels to exert pharmacological effects. The neuroprotective effect of 20(R)-Rg3 was related to attenuating the neuronal apoptosis. The mechanism downregulated the expressions of calpain I and caspase-3 mRNA in hippocampal CA1 region [49] (Table 1).

### 2.4. Brain Protective Effects against Alzheimer's Disease

Alzheimer's disease (AD) is a neurodegenerative senile dementia that usually occurs in the elderly. The main manifestations are memory loss and cognitive deterioration in the early stage. Etiopathogenesis of AD is complex, which is mainly related to amyloid *β*-proteins (A*β*) [75]. How to prevent and cure AD becomes a difficult problem. Ginseng has long been used for the prevention and treatment of AD. And heat-processed ginseng had a better effect than raw ginseng in AD treatment [76].

As we all know, ginsenosides are metabolized into secondary metabolites in the body after oral administration. PPD-type ginsenosides are metabolized to 20-O-*β*-d-glucopyranosyl-20(S)-protopanaxadiol (M1) by intestinal bacteria when taken orally.

The cognitive functions of M1 and ginsenoside Rb1 were explored in AD induced by A*β*_25-35_ i.c.v. injection. After administration of ginsenosides Rb1 and M1, the impaired spatial memory could be improved. The effects of ginsenosides Rb1 and M1 were similar and they could reduce the expression levels of phosphorylated neurofilament-H and synaptophysin in the cerebral cortex and hippocampus of AD mice. In addition, M1 had axonal extension activity and did not have effect on dendrites [50]. The study indicated that AD was closely related to amyloid precursor protein (APP) and presenilin-1 (PS1), which are overexpressed in AD. To explore the anti-AD mechanism of ginsenosides Rg1 and Rg2, a UPLC/MS-based metabolomics investigation was used. The results showed that ginsenosides Rg1 and Rg2 reduced the escape latency in Morris water maze compared to the AD model group. Besides, ginsenosides Rg1 and Rg2 decreased A*β*_1-42_ accumulation in the hippocampus of APP/PS1 mice. In addition, 11 potential biomarkers were detected, which were the metabolism of lysophosphatidylcholines, hypoxanthines, and sphingolipids. This suggested that the anti-AD effect of ginsenosides Rg1 and Rg2 would be partly associated with correcting brain metabolic alterations in AD mice [53]. What is more, ginsenoside Rg1 affected lecithin, amino, and sphingolipid metabolism pathways. However, ginsenoside Rb1 affected lecithin and amino acid but not sphingolipid [54]. These findings suggested that metabolomics study might be a new method to find the therapeutic benefits of ginsenosides in AD treatment.

An AD transgenic mice was used to exam the protective effect of ginsenoside Rg1 on memory performance and synaptic plasticity by using fear conditioning task and western blotting. The results indicated that long-term memory was improved after ginsenoside Rg1 treatment. Ginsenoside Rg1 also did not affect normal diet and weight. Additionally, ginsenoside Rg1 inhibited the levels of C-terminal fragments (CTFs), p-Tau, and *β*-amyloid 1-42 in AD mice. Furthermore, ginsenoside Rg1 could improve the expression of BDNF and tyrosine receptor kinase B (TrkB) in the hippocampus. These results indicated that ginsenoside Rg1 exerted neuroprotective effect by activating the BDNF-TrkB pathway and attenuating the expression of AD-associated proteins [55]. In addition, ginsenoside Rg1 also upregulated the expression of N-methyl-D-aspartate receptor subunit 1 (NR1) and N-methyl-D-aspartate receptor subunit 2B (NR2B) and slowed the formation of neurofibrillary tangles to improve learning and memory abilities in AD mice [56]. Recently, studies have found that sex hormones, especially estrogen, play an important role in the pathogenesis of AD. The rapid decline of estrogen level in postmenopausal women increases their susceptibility to AD. So, a lack of estrogen may be a risk factor for AD. An AD mice were prepared by ovarian steroid deprivation plus d-gal injection. The results suggested that ginsenoside Rg1 not only restored the impaired cognitive performance and promoted spatial learning and memory, but also reduced the A*β*_1-42_ production in AD mice. Besides, ginsenoside Rg1 distinctly increased proportion of *α*-secretase a disintegrin and metallopeptidase domain 10 (ADAM) positive neurons and decreased *β*-secretase *β*-site APP-cleaving enzyme 1 (BACE) positive neurons in hippocampus of AD mice. Additionally, ginsenoside Rg1 attenuated caspase 3 activity, which also reflected the loss of nerve. Interestingly, ginsenoside Rg1 and 17-*β*-estradiol have similar effects on improving cognitive performance and regulating the APP pathway in AD rats [57]. Besides, chronic restraint stress could accelerate the generation and progression of AD. Ginsenoside Rg1 protected against ROS oxidative damage and inhibited NOX2, p47phox, and RAC1 expression [58]. The relationship between ginsenoside Rg1 and endoplasmic reticulum-stress mediated apoptotic pathway was studied in AD rats. The results showed that ginsenoside Rg1 reduced the accumulation of neurofibrillary tangles and the number of terminal deoxynucleotidyl-transferase-mediated dUTP nick end labeling positive cells in AD mice. What is more, ginsenoside Rg1 inhibited the activation of p-JNK and reduced the expression of glucose-regulated protein 78 (Grp78). Therefore, ginsenoside Rg1 exhibited neuroprotective effect via blocking the endoplasmic reticulum-stress pathway triggered by inositol-requiring enzyme-1 and tumor necrosis factor receptor-associated factor 2 pathway [59].

Pseudoginsenoside-F11 (PF11), one of the components of ginseng, has been found to have neuroprotection and enhanced neuronal activity [77]. PF11 could decrease escape latency in the Morris water maze and block the reduction of step-through latency in the step-through test on the A*β*_1-42_-treated mice and APP/PS1 mice. Besides, PF11 inhibited APP expression and A*β*_1-42_ production in the hippocampus and cortex of APP/PS1 mice. PF11 distinctly increased the activities of SOD and glutathione peroxidase and decreased the amount of MDA. In addition, PF11 downregulated the expression of c-Jun N-terminal protein kinase 2 (JNK 2), p53, and cleaved caspase 3. So, the recognition improvement effect of PF11 might be related to anti-inflammation, antiapoptosis, and the inhibitory effect on amyloidogenesis [60]. Similarly, ginsenoside R1 was found to have protective effect against A*β* neurotoxicity [78].

Ginsenoside Rb1 also reduced the A*β*_1-42_ existence in cortex and hippocampus regions and improved the learning ability of AD mice. As is known to all, cyclooxygenase-2 (COX-2), IkB-*α*, and nNOS are the markers in neuroinflammation. Ginsenoside Rb1 could decrease the COX-2 positive cells and IkB-*α* positive cells and increase the nNOS positive cells in hippocampus compared to model group. So, the neuroprotection effect of ginsenoside Rb1 might be associated with anti-inflammation markers in the hippocampus of AD mice [51]. What is more, ginsenoside Rb1 was absorbed rapidly compared with ginsenosides Re and Rg1 [79]. Therefore, ginsenoside Rb1 may be more effective than other ginsenosides. Besides, ginsenoside Rb1 also exhibited a higher scavenging activity against neurotransmitters. For example, ginsenoside Rb1 has a higher scavenging activity against ONOO^−^ than ginsenosides Rb2, Rc, Re, Rg1, and Rg3 [52].

An AD model was made by injecting aggregated *β*-amyloid peptide 1-40 into hippocampus bilaterally. Findings showed that ginsenoside Rd could ameliorate learning and memory ability and reduce the neuronal death and loss in the CA1 regions of hippocampus in AD mice. In addition, ginsenoside Rd decreased the expression of Ibal-1, glial fibrillary acidic protein (GFAP), IL-1*β*, IL-6, TNF-*α*, caspase-3, and S100*β* mRNA. Besides, ginsenoside Rd increased IL-10 and HSP70 mRNA expression levels [61]. In addition, ginsenoside Rd also inhibited the expression of NF-kB p65, which was the important marker of NF-kB pathway [62]. These results indicated the neuroprotective effect of ginsenoside Rd might be associated with anti-inflammation, antioxidation, and antiapoptosis.

Acetylcholinesterase (AChE) and butyrylcholinesterase (BChE) play a significant role in AD. The inhibition of AChE and BChE provides additional benefits in AD treatment. Ginsenosides Rb1, Rb2, Rc, Re, Rg1, and Rg3 have significant inhibitory effect against AChE and BChE. Among these ginsenosides, ginsenoside Re could better inhibit AChE activity [52]. Some studies found that ginsenosides exerted neuroprotective effects by affecting neurotransmitters in AD. For instance, ginsenosides could increase the levels of *γ*-aminobutyric acid, acetylcholine, and dopamine and decrease glutamate and aspartic acid levels in the hippocampus and cortex. Besides, ginsenosides could increase glycine and serotonin levels in the blood [80]. This provides a new way to treat AD (Table 2).

### 2.5. Brain Protective Effects against Parkinson's Disease

Parkinson's disease (PD) is a common neurodegenerative disease of the central nervous system, which is characterized by abnormal movements [81]. The typical neuropathy is the degeneration and deletion of dopaminergic neurons in substantia nigra of midbrain, but its pathogenesis is not yet clear. To study the mechanism of ginsenosides in PD is conducive to better treatment of PD.

As is known to all, the most important feature of PD is movement disorders. Animal models were prepared by using the neurotoxin 1-methyl-4-phenyl-1, 2, 3, 6-tetrahydropyridine (MPTP). The accelerating rotarod, wire suspension, and pole tests were used to test the motor behaviors of mice. The results showed that mice treated with MPTP spent more time in wire suspension test and pole test. However, ginsenoside Rg1 alleviates the MPTP-induced motor deficit. Besides, ginsenoside Rg1 attenuated the decrease of antityrosine hydroxylase (TH) expression and the increase of *α*-synuclein expression in the substantia nigra and striatum. And ginsenoside Rg1 could alleviate the loss of dopaminergic neurons in treated MPTP mice. So, ginsenoside Rg1 may be a potential therapeutic drug against PD by decreasing *α*-synuclein levels in the SN and striatum [63, 64]. In addition, ginsenoside Rg1 could alleviate the mortality of rats and decrease the level of TNF-*α*, IFN-*γ*, IL-1*β*, and IL-6 in the substantia nigra pars compacta (SNpc) in MPTP-treated mice [65, 66]. What is more, T cell subsets would be changed in MPTP-treated mice. Ginsenoside Rg1 not only could increase CD3+CD4+ T cells and percentage of CD4+CD25+Foxp3+ regulatory T cells, but also decrease CD3+CD8+ T cells in PD mice. Ginsenoside Rg1 also inhibited the activation of microglia in the SNpc region [66]. 6-Hydroxydopamine (6-OHDA) was infused into the medial forebrain bundle to damage the nigrostriatal dopamine pathway of ovariectomized rats. JB-1, the antagonist insulin-like growth factor-I receptor (IGF-IR), and ginsenoside Rg1 were tested in PD model. The finding indicated that ginsenoside Rg1 ameliorated the rotational behavior induced by apomorphine, but JB-1 blocked this effect. Additionally, ginsenoside Rg1 improved the gene expressions of TH, the dopamine transporter, and Bcl-2 after 6-OHDA lesions, but JB-1 is still against these effects of ginsenoside Rg1. Hence, the effect of Rg1 was closely related to its ability to activate the IGF-IR signaling pathway [67]. Besides, the neuroprotective effects of ginsenoside Rg1 were also blocked by Dickkopf-1, which is antagonist of Wnt signaling pathway. Wnt/*β*-catenin signaling pathways are thought to play an important role in PD [82]. Therefore, the effects of ginsenoside Rg1 were closely related to Wnt/*β*-catenin signaling pathway [68] (Table 2).

## 3. Conclusion 

Ginseng is a traditional Chinese medicine. Modern pharmacological studies have shown that it has a regulatory effect on the central nervous system. Ginseng can strengthen the cerebral cortex excitatory and inhibitory processes and reduce the fatigue of the brain process. The protective effect of ginseng is mainly due to the role of ginsenosides. Recently, studies have shown that ginsenosides have effects on the nervous system, the cardiovascular system, and the immune system. And few studies have found that ginsenosides are toxic. Although there are many studies on ginsenosides in nervous system diseases, the specific mechanism is still in the basic stage. From what has been discussed above, the neuroprotective mechanism of ginsenosides mainly is mainly related to its anti-inflammatory, antioxidation, antiaging, nerve growth factor, other cytokines, and various signaling pathways. In this paper, we summarize the mechanism of ginsenosides in several neurological diseases in order to explain the neuroprotective mechanism of ginsenoside and provide a useful thinking of ginsenosides in clinical application.

## Figures and Tables

**Table 1:** Pharmacological activity and mechanism of ginsenosides on epilepsy, depression, and cerebral ischemia reperfusion injury.

Pharmacological effects	Ginsenosides	Mechanism	References
Antiepilepsy	Total ginsenosides	Adenosine A_2A_ receptor ↓	[21]

	Rg3	NMDA receptor-mediated Ca^2+^ ↓	[20]

Antidepressant-like effects	Rg1	PKA and CREB phosphorylation, the expression of BDNF, hippocampal neurogenesis ↑	[31, 32]

	Total ginsenosides	IL-1*β*, IL-6, TNF-*α*, IDO ↓ ACTH, CORT ↑	[35, 36]

	Rb1	5-HT_2A_ receptors ↑	[34]

	Rb3	BDNF levels ↑ 5-HT ↑↓	[28]

	Re	BDNF mRNA expression ↑ TH, CRF ↓	[27]

	CK	5-HT_2A_ receptors, NA↑ ACTH, corticosterone level ↓	[29, 34]

	Rg3	BDNF signaling pathway, NA↑ ACTH, corticosterone level ↓	[29, 30]

Protection against cerebral ischemia reperfusion injury	Rg1	Bcl-2, Trx-1, SOD-1, PKB/Akt, p-NF-kB p65, HSP70 ↑ Bax, aquaporin 4 expression, IL-1*β*, TNF-*α*, HMGB1, cleaved caspase-3, cleaved caspase-9, RAGE, PAR-1, Ca^2+^ overload, NF-kB, JAK1/STAT1 signal pathways↓	[37–42]

	Rb1	MDA, NF-k*β*, JAK1/STAT1 signal pathways, TNF-*α*, IL-6, LC3, Beclin 1↓ Trx-1, SOD-1, PKB/Akt, HSP70 ↑	[42–45]

	Rd	VEGF, BDNF, PI3K/Akt, ERK1/2 pathways ↑ phosphorylation of NMDAR 2B subunit ↓	[46, 47]

	Re	MDA ↓H^+^-ATPase ↑	[48]

	Rg3	Calpain I, caspase-3 ↓	[49]

**Table 2:** Pharmacological activity and mechanism of ginsenosides on Parkinson and Alzheimer's disease.

Pharmacological effects	Ginsenosides	Mechanism	References
Anti-Alzheimer's disease	Rb1	Phosphorylated NF-H and synaptophysin, ONOO^−^, A*β*_1-42_, COX-2, IkB-*α*↓ nNOS↑	[50–52]

	Rg1	A*β*_1-42_ accumulation, CTFs, p-Tau, and *β*-amyloid 1-42, BACE 1, NOX2, p47phox, RAC1, NFTs, TUNEL-positive cells, p-JNK ↓ BDNF, TrkB, NR1, NR2B, ADAM 10 ↑	[53–59]

	Rg2	A*β*_1-42_ accumulation	[53]

	F11	A*β*_1-42_ accumulation, APP expression, MDA, JNK 2, p53, cleaved caspase 3↓ SOD, GSH-Px ↑	[60]

	Rd	Ibal-1, GFAP, IL-1*β*, IL-6, TNF-a, S100*β*, IL-10, HSP70, GSSG/GSH ratio, caspase-3, NF-kB p65 ↓	[61, 62]

	Re	AChE ↓	[52]

Anti-Parkinson's disease	Rg1	MPTP-induced motor, TH, CD3+CD4+ T cells, TH, DAT, Bcl-2, Wnt/*β*-catenin signaling pathway ↑ *α*-synuclein, TNF-*α*, IFN-*γ*, IL-1*β*, IL-6, CD3+CD8+ T cells↓	[63–68]
